# A Molecular-Sieving Interphase Towards Low-Concentrated Aqueous Sodium-Ion Batteries

**DOI:** 10.1007/s40820-024-01340-5

**Published:** 2024-03-04

**Authors:** Tingting Liu, Han Wu, Hao Wang, Yiran Jiao, Xiaofan Du, Jinzhi Wang, Guangying Fu, Yaojian Zhang, Jingwen Zhao, Guanglei Cui

**Affiliations:** 1 Qingdao Industrial Energy Storage Research Institute, Qingdao Institute of Bioenergy and Bioprocess Technology, Chinese Academy of Sciences, Qingdao, 266101 People’s Republic of China; 2 Center of Materials Science and Optoelectronics Engineering, University of Chinese Academy of Sciences, Beijing, 100049 People’s Republic of China; 3 School of Chemical Engineering and Advanced Materials, The University of Adelaide, Adelaide, SA 5005 Australia; 4 Shandong Energy Institute, Qingdao, 266101 People’s Republic of China; 5 Qingdao New Energy Shandong Laboratory, Qingdao, 266101 People’s Republic of China

**Keywords:** Molecular sieving effect, Electrode coatings, Aqueous sodium ion batteries, Dilute aqueous electrolytes

## Abstract

Aqueous sodium-ion batteries are known for poor rechargeability because of the competitive water decomposition reactions and the high electrode solubility. Improvements have been reported by salt-concentrated and organic-hybridized electrolyte designs, however, at the expense of cost and safety. Here, we report the prolonged cycling of ASIBs in routine dilute electrolytes by employing artificial electrode coatings consisting of NaX zeolite and NaOH-neutralized perfluorinated sulfonic polymer. The as-formed composite interphase exhibits a molecular-sieving effect jointly played by zeolite channels and size-shrunken ionic domains in the polymer matrix, which enables high rejection of hydrated Na^+^ ions while allowing fast dehydrated Na^+^ permeance. Applying this coating to electrode surfaces expands the electrochemical window of a practically feasible 2 mol kg^–1^ sodium trifluoromethanesulfonate aqueous electrolyte to 2.70 V and affords Na_2_MnFe(CN)_6_//NaTi_2_(PO_4_)_3_ full cells with an unprecedented cycling stability of 94.9% capacity retention after 200 cycles at 1 C. Combined with emerging electrolyte modifications, this molecular-sieving interphase brings amplified benefits in long-term operation of ASIBs.

**Supplementary Information:**

The online version contains supplementary material available at 10.1007/s40820-024-01340-5.

## Highlights

- A molecular-sieving electrode coating towards low-concentrated aqueous sodium-ion batteries is constructed by applying a composite of NaX zeolite and NaOH-neutralized Nafion.
- Resulting from a molecular sieving effect of zeolite channels and size-shrunken ionic domains in Nafion, the as-prepared coating layer reject hydrated Na^+^ ions and allow fast dehydrated Na^+^ permeance.
- 200 cycles of Na_2_MnFe(CN)_6_//NaTi_2_(PO_4_)_3_ full cells can be achieved in a practically feasible 2 m aqueous electrolyte.

**Supplementary Information:**

The online version contains supplementary material available at 10.1007/s40820-024-01340-5.

## Graphical abstract

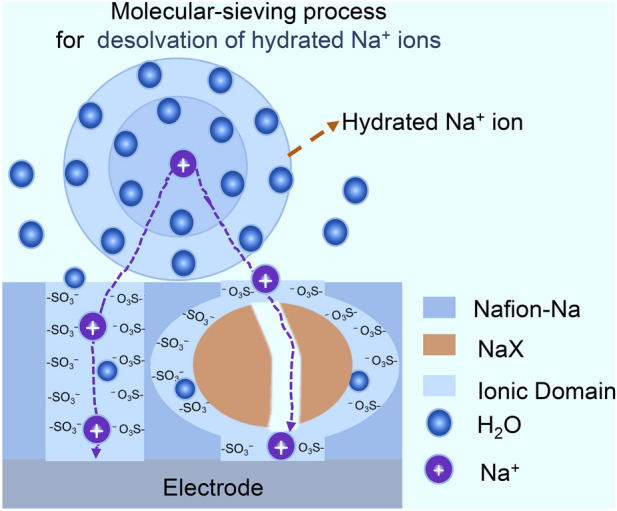

## Introduction

With the demand for large-scale energy storage technologies ever increasing, rechargeable aqueous batteries, especially those using abundant earth elements, such as sodium, as mobile charge carriers, have been actively pursued [1–4]. Unfortunately, the electrochemical reactive nature and the strong solvation ability of water lead to the limited electrochemical operation windows of aqueous electrolytes and the irreversible dissolution of Na^+^-storage electrodes, respectively. The development of aqueous sodium-ion batteries (ASIBs) has long been afflicted by non-competitive energy density (< 30 Wh kg^–1^) and poor cyclability [5, 6].

The *in-situ* formation of anion-derived fluoride-rich solid electrolyte interfaces (SEIs) on anodes in salt-concentrated electrolytes can suppress the hazardous hydrogen evolution reaction (HER), which makes “water-in-salt” electrolytes (WISEs) a promising solution for improving rechargeability of ASIBs [7–9]. However, using electrolytes with excess salts, especially fluorine-containing ones, leads to cost and environmental concerns [10]. The application scope of WISEs is also limited by the relatively poor solubility of fluorinated sodium salts in water [11]. Owing to the non-negligible presence of free water molecules, even saturated aqueous solutions of fluorinated sodium salts (such as 9 m sodium trifluoromethane sulfonate, NaOTF) can hardly tackle the dissolution of Na^+^-storage electrodes and sustain the expected effect from protecting possible SEIs, especially at the high voltage range [12, 13]. Other strategies through introducing organic co-solvents into aqueous electrolytes, such as urea [14] and ethanol [15], which can alter the bulk hydrogen-bonding network [16], were also applied to suppress the water activity and improve the sustainability of SEIs; however, the limited safe and cost benefits make it challenging to evaluate their practical effectiveness.

Given the unstable nature of in-situ formed SEIs in aqueous media, reasonable controls over HER cannot solely rely on electrolyte modifications. Engineering *ex-situ* protective interphases between electrodes and electrolytes have attracted much attention in the aqueous battery field [17–21]. In this context, polyethylene oxide (PEO)-based electrode coating layers were proposed [22, 23], but high salt concentrations and additional polymers in electrolytes had to be applied to address the high solubility of PEO. Alternative interfacial strategies that make low-concentrated electrolytes directly feasible for rechargeable operation of ASIBs are necessary if we ever want the originally expected features (i.e., low cost, intrinsic safety, and environmental friendliness) of aqueous electrolytes to be maintained rather than compromised.

As for ASIBs equipped with regular diluent aqueous electrolytes, the electrolyte–electrode interfacial water, which is more susceptible to electrochemical decomposition than the bulk water, consists of hydrogen-bonded and Na^+^ ion hydrated water (Na^+^·H_2_O) portions [24–26]; in particular, the latter tends to delay oxygen evolution reaction (OER) due to the reduction in the charge density of oxygen [10, 27, 28], but has a relatively high activity for HER because of the increased O–H bond length and the reduced O–H bond order (Fig. S1). Therefore, the critical step to suppress HER is to isolate Na^+^·H_2_O from the anode-electrolyte interface. Inspired by the size exclusion sieving process of molecular sieves in the solvation sheath reorganization of cations [27], we extend the application of zeolites to the electrode coating of ASIBs. However, due to their poor film-forming capability, zeolites have not yet been widely applied for electrode modifications. Introducing a polymeric film-forming agent appears to be a more technically and economically feasible solution by which zeolites can be chemically and/or physically immobilized on the electrode surface. Commercially available Nafion (one perfluorosulfonic acid polymer), composed of hydrophilic side chains and hydrophobic main chains, could be a promising candidate [29]. The flexible hydrophobic polytetrafluoroethylene backbone of Nafion provides mechanical support and water resistance; meanwhile, zeolite units could disperse homogenously in the hydrophilic portion to construct uniform coating layers.

Herein, we present a facile strategy by applying a NaX zeolite molecular sieve (abbreviated as NaX) / NaOH-neutralized Nafion (Nafion-Na) composite (Nafion-Na/NaX), as a functional interphase to decouple Na^+^-storage electrodes from a seamless contact with water in ASIBs. Most of challenges originating from water-involved competitive side reactions (i.e., gas production and electrode dissolution/disintegration) can be overcome. The high affinity of hydrophilic regions in Nafion-Na to NaX could weaken the self-aggregation of ionic segments of the polymer matrix. Due to the size exclusion effect, the shrunken ionic channels of Nafion-Na and the sub-nano (0.39 nm) pores of NaX conjointly transport dehydrated Na^+^ ions over hydrated ones, which inhibits HER and suppresses cathode dissolution even in dilute aqueous electrolyte environments. For example, implementing this interphase on both the cathode and anode sides substantially expands the electrochemical stability window (ESW) of 2 m NaOTF from 1.70 to 2.70 V, which is comparable to the benefit brought by highly concentrated aqueous electrolytes (e.g., 9 m NaOTF). The resultant Na_2_FeMn(CN)_6_ (NMF)//NaTi_2_(PO_4_)_3_ (NTP) full cells with interfacial protections can be steadily cycled in 2 m NaOTF, with capacity retention of 94.9% over 200 cycles. Intriguingly, this strategy can also be applicable to other readily available low-concentrated electrolytes, including 1 m Na_2_SO_4_, 2 m NaTFSI, and 2 m NaClO_4_.

## Experimental Section

### Materials

The NMF cathode was synthesized by a simple co-precipitation method [30]. 5 mmol Na_4_Fe(CN)_6_ (Yuanye, Shanghai; 99%) and 15 g NaCl (Macklin; 99.5%) were dissolved into 100 mL deionized water to form solution A. 5 mmol MnCl_2_ (Xianding, Shanghai; 99%) was dissolved into 50 mL deionized water to form solution B. Then, slowly drop solution B (in about 20 min) into solution A under stirring, and then continue stirring for 2 h. The solid phase was obtained by centrifuging the prepared solution and washing 3 times with 30 mL of deionized water. Then, the solid phase was dried and ground into powder, and dried in a vacuum oven at 110 °C for 24 h before use. The NTP anode was purchased from (Klamar, Shanghai; 99%). NaX was synthesized according to a previously reported article [31]. 0.98 g Al(OH)_3_ was added into 2 g NaOH solution (50 wt% in water) and dissolved into transparency solution after stirred at 100 °C. When the solution was cooled down to room temperature, 32.6 g water and 2.9 g NaOH were added and mixed uniformly in an ice-water bath for 1 h. Then 22.35 g Na_2_SiO_3_ solution (32 wt% in water) was added into the solution and stirred fleetly 1 h in ice-water bath. Subsequently, the solution was stirred for another 24 h at room temperature. After that, the solution was hydro-thermal for 8 h at 98 °C. The white product was collected and washed by filtration and dried at 80 °C for 12 h. Nafion-Na was prepared by the following method: the purchased Nafion ((DuPont, D520, 5 wt%) was neutralized by 0.01 M NaOH solution drop by drop. After being purified by a dialysis process, the product was collected by removing the solvent at 60 °C.

### Preparation of Interfacial Coatings

Nafion-Na coating layer was prepared as follows: 0.2 g Nafion-Na was dissolved in a mixed solution of 0.9 g *N*,*N*-Dimethylformamide (DMF) and 0.9 g isopropanol in 60 °C. Then, 30 µL the above solution was dropped on the surface of electrode discs. After removing the solvent at room temperature, the electrode discs were protected by Nafion-Na. The Nafion-Na/NaX coating layer was prepared similarly to the Nafion-Na coating layer except for the solution preparation. 0.2 g Nafion-Na was dissolved in 0.9 g DMF and 0.9 g isopropanol mixed solution in 60 °C. Then, 0.05 g NaX zeolite was added into the above solution and stirred for 0.5 h and ultrasound for 0.5 h. The above procedures were repeated three times to obtain an even mixture.

### Electrochemical Measurements

Electrodes were prepared by mixing active materials, super P and PVdF with a mass ratio of 8:1:1. Then the slurry was blade-coated on a titanium (Ti) foil current collector and dried at 80 °C for 24 h under vacuum. The electrodes were cut into disks with a diameter of 12 mm before use. The resulting average capacity loading of the cathode and the anode is about 0.08 and 0.09 mAh cm^−2^, respectively. The ionic conductivity of electrolytes were measured by electrochemical impedance spectroscopy (EIS) using a glass electrolytic cell with two Pt electrodes. Linear LSV is measured by a three-electrode cell at a scanning speed of 1 mV s^−1^. In the three-electrode cell, glass carbon was used as the working electrode and Pt as the counter electrode, and the Ag/AgCl electrode (sat. KCl, 0.197 V vs. SHE) was used as the reference electrode. Ionic conductivity of the membranes was measured by the two probe AC method on an EC-lab workstation. A circular sample with a diameter of 16.5 mm was placed in a coin cell where it was clamped between two stainless steel electrodes. 20 µL deionized 2 m NaOTF was dropped into membrane prior to the test. The E_a_ was calculated by the reported method [32].

### Characterizations

The IR spectrum is measured on a Fourier transform infrared spectrometer (Bruker VERTEX 70). For transmission electron microscopic (TEM) observations, the Nafion and Nafion-Na/NaX resins were stained with lead ions by immersing the sample in ca. 0.5 M Pb(OAc)_2_ aqueous solution, rinsed with water, dried, and sectioned to 70 nm thickness. The prepared samples were placed on copper grids and analyzed with a Hitachi H-7650 TEM. The small angle X-ray scattering (SAXS) experiments were performed using a Xenocs Nano-inXider instrument. Inductively coupled plasma atomic emission spectrometry (ICP-MS) was used to test the concentration of Mn in the electrolyte after cycling. DEMS batteries were assembled in a glove box filled with argon. DEMS monitored the volatile gases of H_2_ produced during battery operation at room temperature (Hidden HPR40). Energy-dispersive spectrometry (EDS) and scanning electron microscopy (SEM) experiments were conducted with a field emission scanning electron microscope (SEM, Hitachi S-4800). X-ray diffraction (XRD) patterns were recorded using a Bruker-AXS Micro-diffractometer (D8 ADVANCE) with Cu-Kα1 radiation (*λ* = 1.5405 Å).

### Molecular Dynamic Simulations

Molecular dynamic (MD) simulations were performed on the electrolyte mixtures (NaOTf, and H_2_O) to observe the structure changes and diffusion properties of the electrolyte mixtures. First, the optimized electrolyte molecules were packed in a periodic box to construct the bulk systems, the compositions of simulated electrolytes are given in Table S1. Subsequently, all mixture systems were equilibrated by NPT (i.e., isothermalisobaric) MD simulations for 5 ns at 298 K and atmospheric pressure, followed by NVT (i.e., isothermal) MD simulations for 10 ns with a 1 fs time step. All MD simulations were performed using the Forcite code with COMPASSII force field [33]. The temperature was controlled by a Nose–Hoover Langevin (NHL) thermostat and the pressure was controlled by a Berendsen barostat [34, 35]. The Ewald scheme [36, 37] and atom-based cutoff method (i.e., a radius of 15.5 Å) were applied to treat electrostatic and van der Waals (vdW) interactions, respectively. All the partial atomic charges were defined using the COMPASSII force field.

### Density Functional Theory Simulations

Density functional theory (DFT) calculation was performed by Vienna Ab Initio Simulation Package (VASP.5.4.4) [38, 39]. Generalized gradient approximation (GGA) with Perdew–Burke–Ernzerhof (PBE) function was used for exchange–correlation potential [40, 41]. Energy cut-off was set as 450 eV. The van der Waals interaction was simulated by using DFT-D3 correction method. Based on MD mean results, five explicit molecules were added as the first solvation shell besides the NaOTF, while an implicit solvent model was also employed to simulate the second solvation shell and surrounding solvent environment using Polarizable Continuum Model (PCM) provided by VASPSOL [42, 43], where ε_*r*_ = 80 for water system. O–H bond order is acquired through DDEC6 atomic population analysis [15].

## Results and Discussion

### Characterization of Interfacial Coatings

**Fig. 1:**
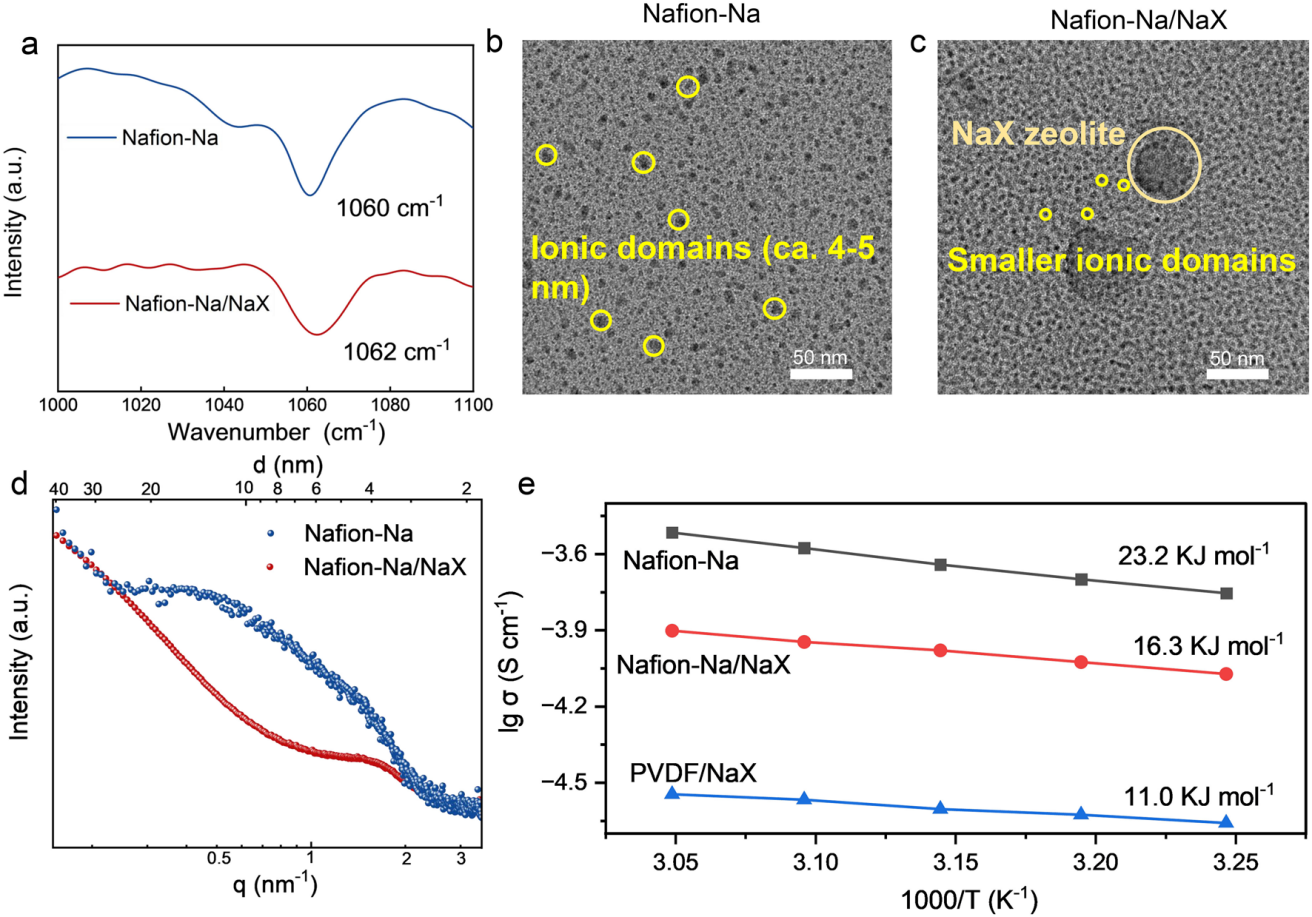
**a** ATR-FTIR spectra of Nafion-Na and Nafion-Na/NaX. **b**, **c** TEM spectra and **d** SAXS patterns of Nafion-Na and Nafion-Na/NaX. **e** Arrhenius plots of ionic conductivity for Nafion-Na, Nafion-Na/NaX and PVDF/NaX membranes (infiltrated with 2 m NaOTF)

The NaX zeolite was synthesized according to previous reports [31] and details are provided in the Supporting Information. After high-temperature (450 °C) vacuum drying, NaX was subjected to infrared characterization (Fig. S6). NaX exhibits typical adsorption bands at around 3400 and 3700 cm^−1^, revealing the presence of hydroxyl groups (-OH) on the skeleton [44]. As shown in Fig. S2, the element composition of NaX was verified by inductively coupled plasma-optical emission spectrometry (ICP-OES, Si/Al/Na = 1.0/2.09/1.66). The XRD pattern of synthesized NaX is well matched with PDF#38–0237 (standard figure); N_2_ adsorption–desorption analyses reveal the high Brunner − Emmet − Teller (BET) surface area (488.06 m^2^ g^−1^) and the pore width (0.39 nm) of obtained NaX (Figs. S3 − S5). To avoid the potential side reactions, the sulfonic acid moieties of Nafion were neutralized by NaOH, and the product was named Nafion-Na. As verified by attenuated total reflectance infrared (ATR-FTIR) spectra (Fig. 1a), after being mixed with NaX, the peak belonging to the –SO_3_^–^ site of Nafion-Na shifted from 1060 to 1062 cm^−1^ along with peak broadening, which is ascribed to the hydrogen-bonding between –SO_3_^–^ of Nafion-Na and hydroxyl groups of NaX [31]. TEM images provide visible evidence for the ionic domains (hydrophilic regions) formed by the aggregation of sulfonic groups and NaX nanoparticles (both are dark regions) in the samples stained with Pb^2+^. The ionic domains in Nafion-Na are around 5 nm in diameter [29] (Fig. 1b), whereas the ionic domain sizes decrease with the incorporation of NaX (Fig. 1c), indicating a less distinct phase separation in the Nafion portion of the Nafion-Na/NaX composite. NaX particles (the dark regions with a diameter of tens of nanometers in Fig. 1c) are evenly distributed in the composite, verifying the polymer-zeolite compatibility. The small angle X-ray scattering (SAXS) profile of Nafion-Na (saturated with 2 m aqueous NaOTF) exhibits a scattering peak of the ionic domains in line with previous reports of Nafion under a fully hydrated condition [29] (Fig. 1d); however, this peak for Nafion-Na/NaX shifts to a higher *q* value, corresponding to a lower *d*-spacing (recognized as the average diameter of ionic domains, about 3.9 nm in width) (*d* = 2π/*q*), confirming the formation of downsized ionic domains. It is clear from ATR-FTIR, TEM and SAXS results that the hydrogen-bonding interaction between Nafion-Na and NaX induces an orientation of -SO_3_^–^ along the NaX surface [45, 46], reducing the concentration of hydrophilic regions in the bulk phase (Fig. S6). This hinders the self-assembly process of -SO_3_^–^ and prevents the formation of large ion channels, as illustrated in Fig. S7, which may bring molecular selective permeance due to the size-sieving effect.

The primary objective of utilizing functional electrode coatings is to achieve preferred target-ion migration, but it can also give rise to considerable interfacial resistance, thereby negatively influencing the rate performance of the battery. To investigate the ion transport behavior in these coatings, the ionic conductivities under different temperatures were evaluated (Fig. 1e). For comparison, the membranes of poly(vinylidene fluoride) (PVdF) doped with NaX (25 wt%, same as that of Nafion-Na/NaX) were prepared and were abbreviated as PVDF/NaX. Considering the ionic insulation of PVdF, the pores of NaX exclusively serve as ion transport channels in PVDF/NaX, resulting in an inferior ionic conductivity (Fig. 1e). The ionic conductivity of Nafion-Na/NaX (8.45 × 10^–5^ S cm^–1^ at 35 °C) significantly exceeds that of PVDF/NaX (2.36 × 10^–5^ S cm^–1^ at 35 °C), providing evidence that both the contracted ionic channels of Nafion-Na and the pores of NaX in Nafion-Na/NaX are involved in ion migration, and is comparable to that of Nafion-Na (1.76 × 10^–4^ S cm^–1^ at 35 °C), promising low resistance to ion transfer. Moreover, among all the samples, PVdF/NaX exhibits the lowest activation energy (*E*_*a*_ = 11.0 kJ mol^–1^, calculated using the Arrhenius equation), signifying low hindrance to ion transport within NaX. This observation supports the preferential transport of ions within the pores of NaX, rather than the Nafion-Na.

**Fig. 2:**
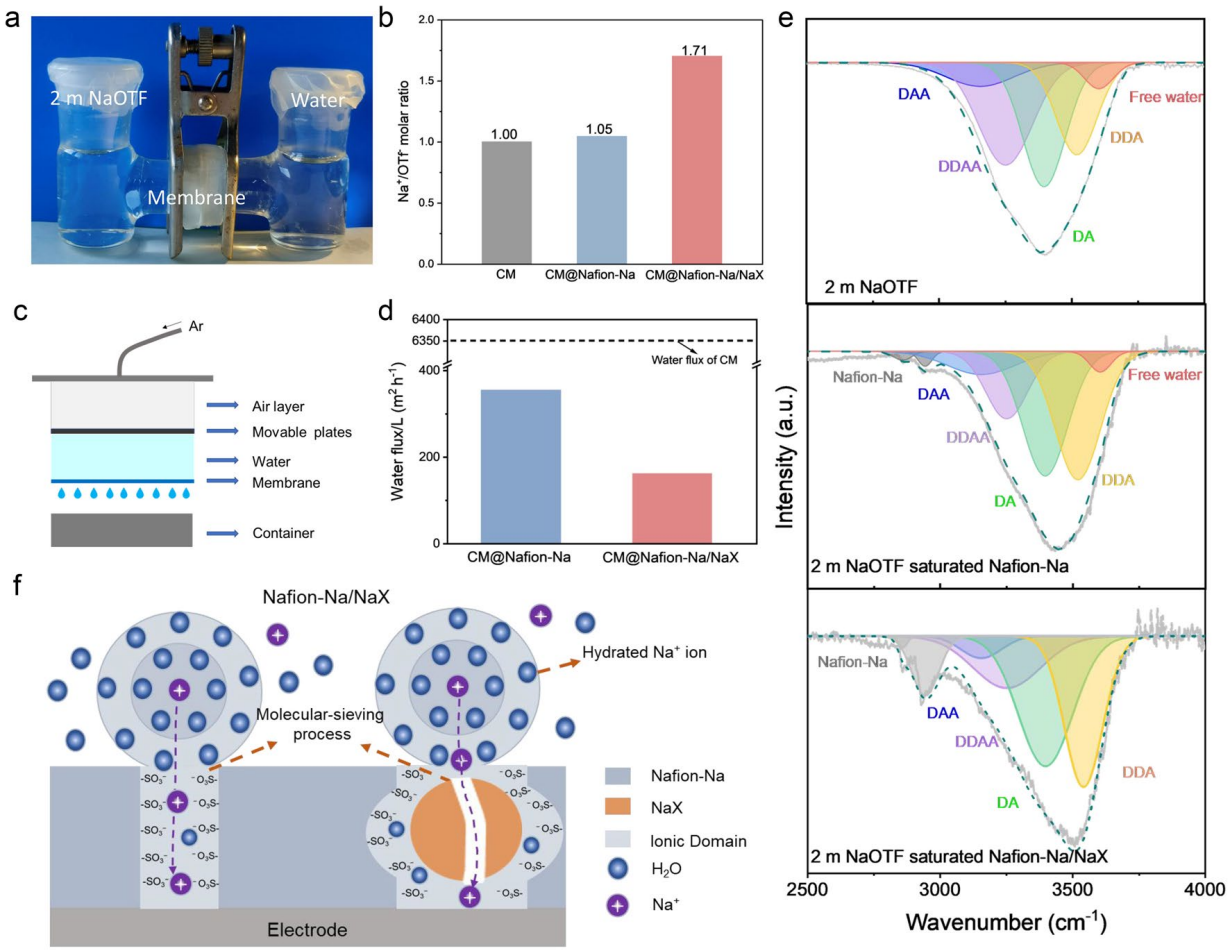
**a** The digital photograph of NaOTF permeability test device and **b** the corresponding Na^+^/OTF^−^ molar ratios of the water side. **c** Schematic of the test device for water permeability. **d** The water flux of CM, CM@Nafion-Na, and CM@Nafion-Na/NaX. **e** ATR-FTIR spectra of 2 m NaOTF, 2 m NaOTF saturated Nafion-Na and Nafion-Na/NaX membranes. **f** Schematic of Nafion-Na/NaX for molecular sieving

To substantiate the sieving effect of the coating layers, an H-typed device (Fig. 2a) was established with porous cellulose membranes (CM), the CM coated with Nafion-Na (CM@Nafion-Na) and Nafion-Na/NaX (CM@Nafion-Na/NaX) as the diaphragms to separate the 2 m NaOTF and water chambers. Due to the concentration gradient, cations and anions in the aqueous salt solution would spontaneously spread to the water side. To maintain electric neutrality, the molar concentration of Na^+^ and OTF^−^ in the water chamber after reaching equilibrium should be the same. However, the negatively charged ion transport channels of Nafion-Na/NaX would hinder permeance of OTF^−^ because of both size selectivity (~ 0.68 nm for OTF^−^, much larger than that of Na^+^, 0.20 nm) (Fig. S8) and electrostatic repulsion. Therefore, the sieving effect of the membrane can be translated by an increase of Na^+^/OTF^−^ molar ratio. ICP-OES tests collected the data of cation and anion concentrations in pure water sides after 2 h (Fig. S9). The presence of Nafion and Nafion-Na/NaX coated cellulose membranes results in much lower cation and anion concentrations compared with the uncoated CM. However, the cation/anion ratios (Fig. 2b) for the uncoated CM and CM@Nafion-Na systems are close to 1, indicating that their large ion channels exert no selectivity for Na^+^ and OTF^−^. In sharp contrast, the Na^+^/OTF^−^ ratio for Nafion-Na/NaX case is as high as 1.71, evidencing the size-sieving effect of the ion migration channels: penetration proclivity for Na^+^ and strong resistance to OTF^−^.

This size-sieving effect of the Nafion-Na/NaX membrane may be beneficial for exclusion of hydrated Na^+^ ions possessing a high HER tendency. Indeed, in 2 m NaOTF, according to molecular dynamics (MD) simulations, Na^+^ is typically surrounded by two concentric solvent shells (namely the first and second solvation sheaths) with diameters of 0.46 and 0.88 nm, respectively (Figs. S10, S11) [47], which are comparable to or even larger than that of OTF^−^.

In terms of the suppression of HER in ASIBs, the coating layers are expected to play a role in preventing the electrode from direct contact with water in the bulk electrolyte, and their water permeability were thereafter evaluated (Fig. 2c, d). As compared to plain CM, both CM@Nafion-Na and CM@Nafion-Na/NaX exhibit significantly decreased water permeance, due to the dense coatings. Owing to the reduced size of hydrophilic regions in Nafion-Na/NaX, CM@Nafion-Na/NaX exhibits higher water rejection (water flux: 163 L m^–2^ h^–1^) compared with CM@Nafion-Na (water flux: 356 L m^–2^ h^–1^). The interaction between the coating layers and water was investigated by ATR-FTIR (Fig. 2e). The O–H stretching band of water can be deconvoluted into several Gaussian peaks corresponding to hydrogen-bonded water with different donor (D) and acceptor (A) hydrogen bonds, which are engaged in DAA, DDAA, DA, DDA hydrogen-bonding and free O–H [22, 48, 49]. Compared with the that of 2 m NaOTF bulk electrolyte, an overall blue shift in the O–H stretching vibration peak observed in the Nafion-Na membrane saturated with 2 m NaOTF could be attributed to the hydrogen-bonding interaction between water and the -SO_3_^–^ groups of Nafion-Na. In the case of the Nafion-Na/NaX membrane saturated with 2 m NaOTF, the hydrogen-bonding interactions are further enhanced, as evidenced by the disappearance of the free O–H peak and a more pronounced blue shift in the overall O–H stretching vibration peak. This is most likely attributed to two factors. (1) The shrunken hydrophilic domains facilitate intimate contact between water and –SO_3_^–^ groups of Nafion-Na; (2) the hydroxyl groups on the NaX surface also contribute to the formation of hydrogen bonds (Fig. S12). The strong hydrogen-bonding environments restricts the permeation of hydrated ions and water [16], but could facilitate the desolvation of hydrated Na^+^ ions [31], resulting in the preferred Na^+^ transmembrane transport. In light of the above results, we propose a scenario, illustrated in Fig. 2f, to account for the species-sieving effect of Nafion-Na/NaX.

### Effect of Interfacial Layers on Electrochemical Properties

**Fig. 3:**
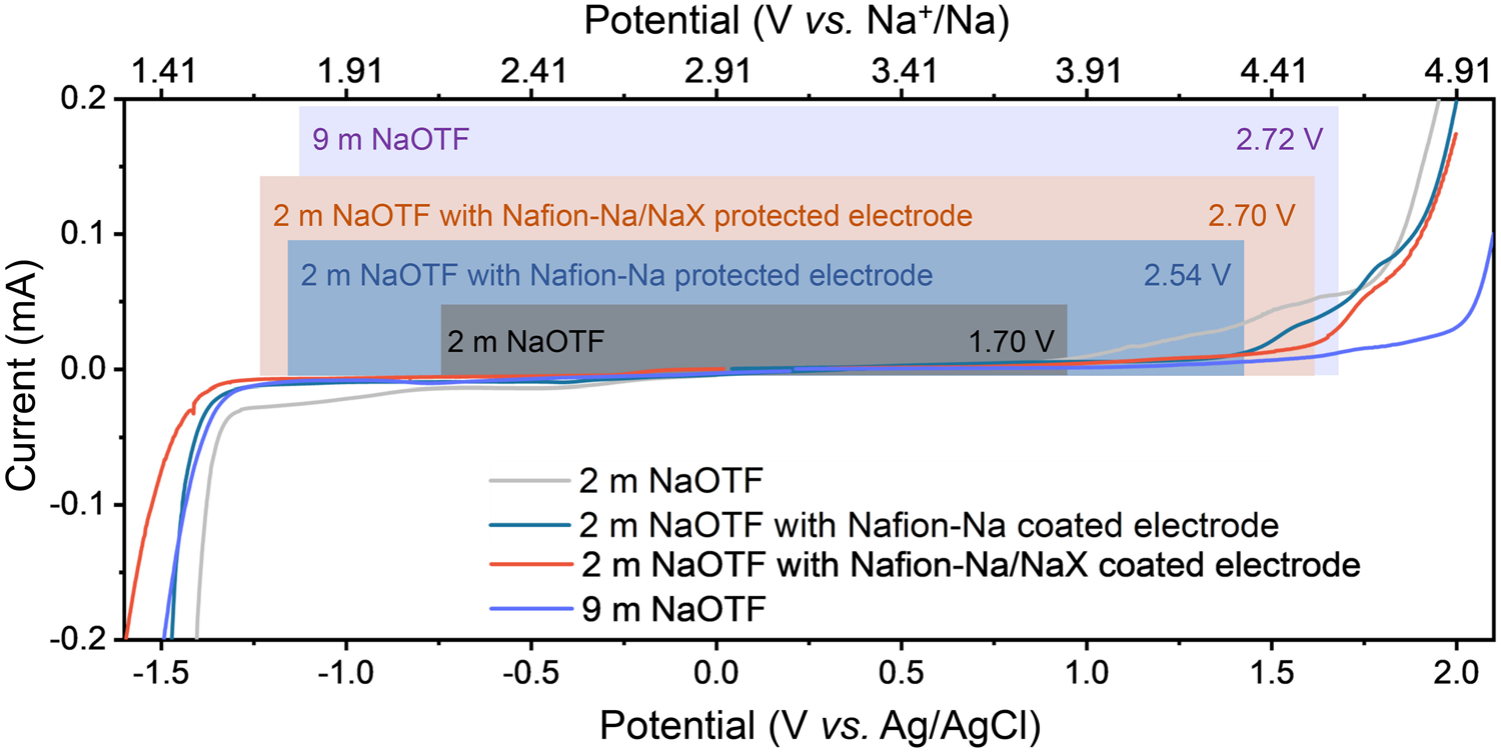
ESWs of 9 m NaOTF on the unprotected working electrode and 2 m NaOTF, on unprotected, Nafion-Na protected, and Nafion-Na/NaX protected working electrodes at a scan rate of 1.0 mV s^−1^

To demonstrate the sieving effect of Nafion-Na/NaX, we first coated Nafion and Nafion-Na/NaX on the surface of glassy carbon electrodes and then assembled three-electrode cells to test the ESW of a practically feasible aqueous electrolyte (2 m NaOTF). As shown in Fig. 3, when the glassy carbon electrode is protected by Nafion-Na or Nafion-Na/NaX, the HER potential of 2 m NaOTF is pushed well beyond the reduction limit of water. Surprisingly, the interfacial protection not only suppresses HER but also delays the anodic limit of aqueous electrolytes. The Nafion-Na protective layer enlarges the ESW to 2.54 V; a further widened ESW of ~ 2.70 V can be achieved by the Nafion-Na/NaX protection. Moreover, the enhanced electrochemical performance achieved with the Nafion-Na/NaX protection is even comparable to that obtained by the salt-concentrated strategy (i.e., using 9 m NaOTF). The delay in the onset potential of OER may be attributed to the blocking effect of the coating on water [50].

### Electrochemical Performance of Full Cells Equipped with Interfacial Layers

**Fig. 4:**
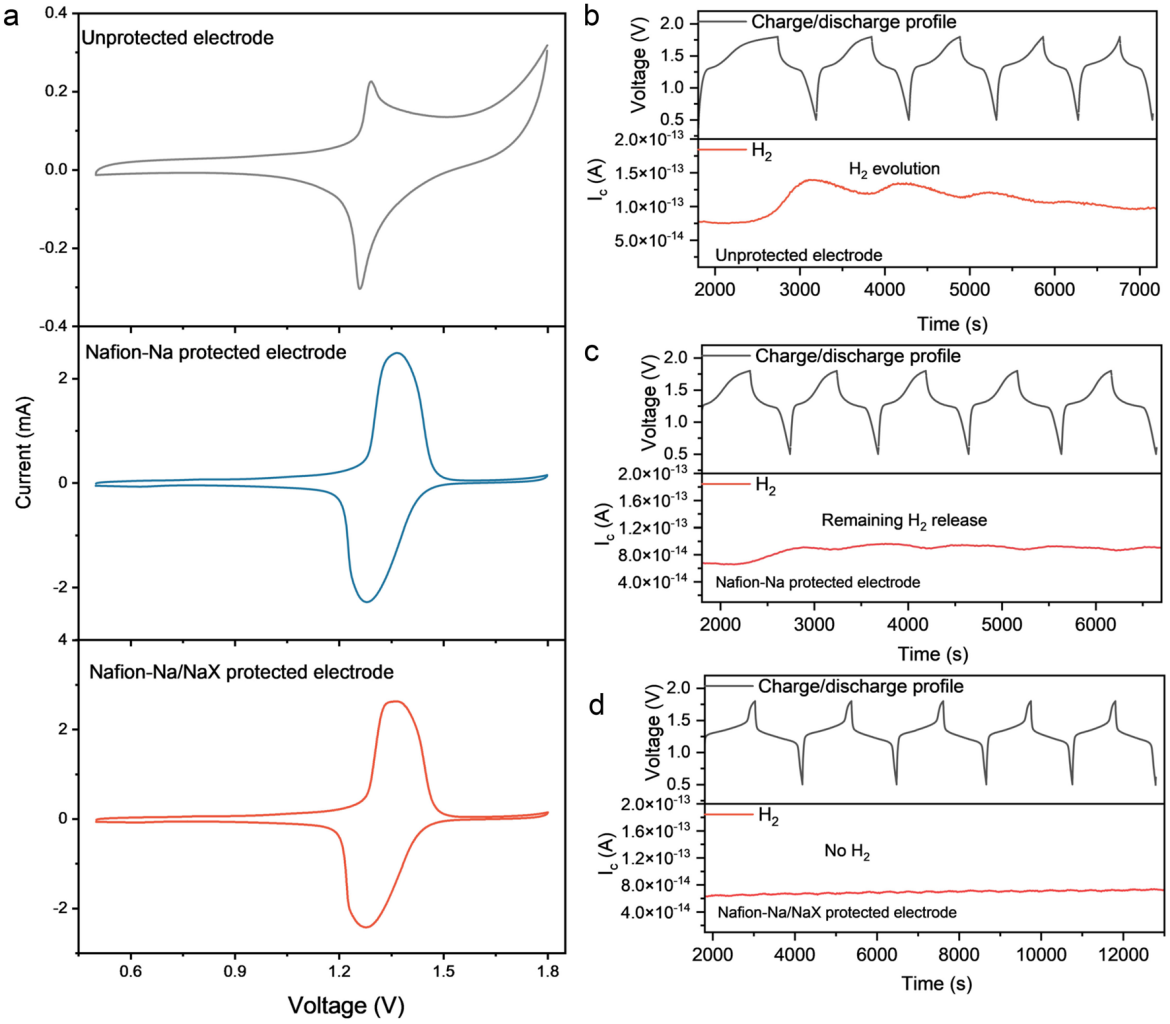
**a** CV curves of NMF//NTP full cells equipped with unprotected, Nafion-Na protected and Nafion-Na/NaX protected electrodes using 2 m NaOTF (performed at 1 mV s^−1^). Voltage profiles and H_2_ signals of NMF//NTP cells in the 2 m NaOTF electrolyte with **b** unprotected, **c** Nafion-Na protected and **d** Nafion-Na/NaX protected electrodes at 140 mA g^−1^

Full cells using NMF cathodes and NTP anodes were assembled to evaluate the viability of the molecular-sieving interphase. The coating process involves depositing a precursor solution of Nafion-Na/NaX in a mixture of DMF and isopropanol onto the electrode surface. The contact angle between the droplet of the precursor solution and the electrode surface is only 33.47° (Fig. S13), indicating excellent wetting properties between them. Thus, the precursor solution could automatically spread on the electrode surface to form a uniform coating layer, with a controlled thickness of ~ 9.3 µm (Fig. S14). Cycling voltammetry (CV) profiles (Fig. 4a) show that the baseline cell with unprotected electrodes is intrinsically susceptible to water-induced side reactions as expected, according to a sharp current increase above 1.6 V. In contrast, no side-reaction signals appeared at the voltage range of 0.5 − 1.8 V for the cells utilizing electrodes protected by Nafion-Na and Nafion-Na/NaX, respectively; meanwhile, the well-defined redox peaks of Fe^3+^/Fe^2+^ demonstrate unobstructed Na^+^ permeance across coatings. To quantitatively analyze HER, the in-situ monitoring gas evolution was conducted within the charge–discharge voltage range same as that for CV tests by the differential electrochemical mass spectrometry (DEMS) (Fig. 4b–d). Upon increasing the voltage to 1.6 V for unprotected electrodes, a notable rise in the H_2_ signal was observed, in line with the CV results. The cell equipped with Nafion-Na-covered electrodes displayed reduced H_2_ evolution. However, full suppression of HER was not attained, and the HER onset potential persisted at around 1.6 V due to the ongoing accessibility of hydrated Na^+^ ions to the anode. Importantly, for the cell with Nafion-Na/NaX coated electrodes, no visible H_2_ signal can be detected upon cycling, highlighting that the molecular sieving effect can essentially suppress HER while ensuring reversible Na^+^ permeance. Due to the relatively sluggish kinetics of OER, it is difficult to detect oxygen signals even for cells cycled without molecular-sieving interphases (Fig. S15) [51].

**Fig. 5:**
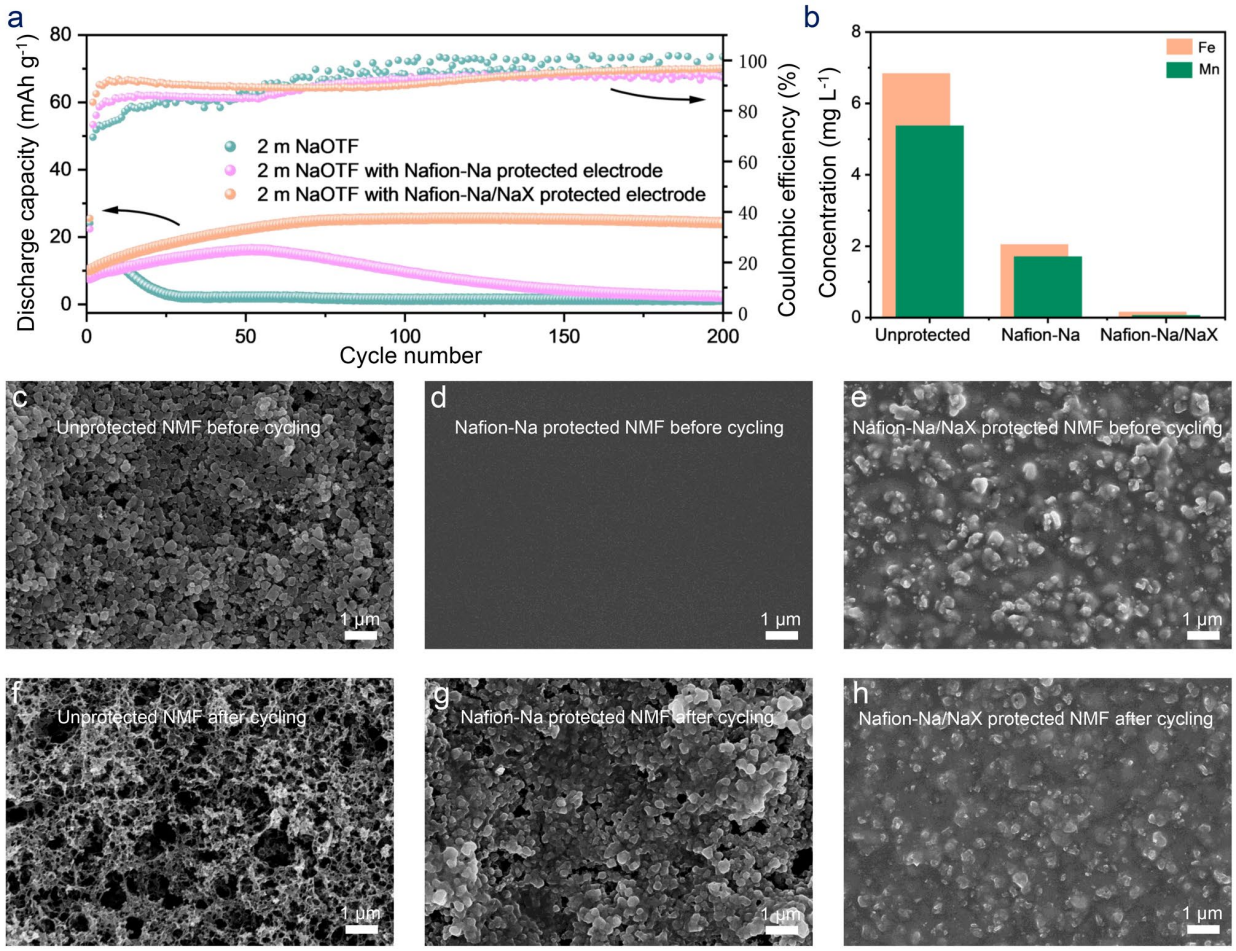
**a** Cycle performance of the NMF//NTP full cells in 2 m NaOTF with unprotected, Nafion-Na protected, and Nafion-Na/NaX protected electrodes at 140 mA g^−1^ (based on the mass of NMF). **b** ICP-OES results of Mn and Fe contents in electrolytes of full cells after 200 cycles. SEM images of unprotected, Nafion-Na protected, and Nafion-Na/NaX protected cathodes **c**–**e** before and **f–h** after 200 cycles

The positive attributes of the molecular-sieving interphase concerning enlarged ESWs and suppressed gas evolution can be anticipated to bring a significant enhancement of cyclability to ASIBs. The cycling performance of the aqueous NMF//NTP full cells was measured within 2 m NaOTF as electrolyte (Fig. 5a); a negative to positive (N–P) capacity ratio is fixed at 1.1 in all tested cases**.** The control cell with unprotected electrodes underlining the intrinsic irreversibility of ASIBs caused by water-induced side reactions given severe capacity degradation after only ten operation cycles. With the electrodes being modified by Nafion-Na alone, both the onset of capacity ‘rollover’ and complete cell failure are extended, but the cell hardly survived the long-cycling trial. More encouraging still is what happens when the additional NaX was adopted. The cell with Nafion-Na/NaX protection made it to over 200 cycles before dropping below 94.9% (relative to the highest capacity during cycling), which is indeed impressive since this cyclability is even better than that provided by using a saturated aqueous NaOTF (9 m) electrolyte (Fig. S16). The Nafion-Na/NaX membrane enables the dilute 2 m NaOTf electrolyte to support stable cycling of NMF//NTP cells with an output voltage of > 1.0 V. The sub-100% Coulombic efficiency of the battery is probably attributed to the slow ingress of a small amount of water molecules through the Nafion-Na/NaX protection to the electrode region. During the initial 50 cycles, we noticed that the cell capacity increased gradually and eventually stabilized. This phenomenon could be ascribed to the slow activation process and/or the enhancement in utilization of active materials [28].

Importantly, such improvements in ASIB cyclability are elucidated to be only reliable when using the Nafion-Na/NaX protection on both cathode and anode surfaces (Fig. S18). In addition to the merit of the suppressed H_2_ evolution, the positive attribute of the artificial interphase against the solubility of the Na^+^-storage cathodes can be rationalized [52]. As expected, the ICP-OES tests of the cycled electrolytes (Fig. 5b) show that Fe and Mn dissolution amounts of NMF covered by Nafion-Na/NaX (0.16 mg L^–1^ for Fe and 0.06 mg L^–1^ for Mn) are much less than those of bare NMF (6.84 mg L^–1^ for Fe and 5.37 mg L^–1^ for Mn) and NMF covered by Nafion-Na (2.05 mg L^–1^ for Fe and 1.71 mg L^–1^ for Mn). Additionally, it is clear from the SEM imaging analysis (Fig. 5c-h) that, in comparison to its pre-cycling state, the cycled Nafion-Na protected NMF becomes visibly bare, indicating the dissolution of Nafion-Na in 2 m NaOTF upon cycling. Compared with unprotected and Nafion-Na coated cathodes, the cathode protected by Nafion-Na/NaX maintained a more intact microscale morphology after cycling. In addition, there is no noticeable dissolution of the coating during the battery cycling process, as observed by unchanged thickness after 200 cycles (Fig. S17). This is attributed to the presence of insoluble NaX, which serves as an anchor, preventing the dissolution of Nafion-Na. Furthermore, the enhanced Mn and Fe signals and the decreased S signal of the Nafion-Na protected cathode after cycling, as observed in the results of energy dispersive spectroscopy (EDS) mapping (Figs. S19, S20), manifest the interphase solubility issue when only Nafion-Na was applied. The EDS mapping results of the cathode protected by Nafion-Na/NaX show minimal changes after cycling, underscoring the exceptional stability of Nafion-Na/NaX.

We evaluated the generality of this protective interphase with other diluted aqueous electrolytes (Fig. S21). As expected, the benefits of Nafion-Na/NaX-protected electrodes in suppressing water-induced side reactions while extending the ESW of the dilute electrolyte can be also distinguished for 2 m NaClO_4_, 2 m sodium bis(trifluoromethanesulfonyl)imide (NaTFSI) and 1 m Na_2_SO_4_. In addition, from a practical point of view, Nafion-Na can also be replaced by other cost-effective film-forming polymers with both hydrophobic backbones and hydrophilic side chains (-SO_3_^−^), such as sulfonated poly(ether ether ketone) (SPEEK) (Fig. S22a). Specifically, the SPEEK/NaX (a mixed membrane composed of SPEEK and NaX) protection elevates the ESW of the 2 M NaOTF electrolyte to 2.81 V, even slightly wider than that obtained from the Nafion-Na/NaX case (Fig. S22b), and consequently further enhances the cyclic stability of the NMF//NTP cell (Fig. S22c). More importantly, this facile interphase strategy is compatible with currently accepted electrolyte modifications (e.g., WISE), aiming at achieving further gains in the rechargeability of ASIBs. As demonstrated in Fig. S18, the integration of Nafion-Na/NaX protected electrodes with 9 m NaOTF resulted in enhanced battery stability, substantiating the significant potential of interfacial strategies in expediting the advancement of high-performance aqueous batteries. Moreover, through a statistical analysis of reported studies on electrode modification in ASIBs (Table S2), it is evident that Nafion-Na/NaX represents the first instance of simultaneously safeguarding both the cathode and anode, enabling stable cycling of full cells in dilute electrolytes with the highest capacity retention (94.9%) and Coulombic efficiency (96.8%).

## Conclusions

In summary, based on the fact that the rechargeability of current ASIBs overly relies on the use of concentrated electrolytes, we here report an artificial electrode interface strategy by employing a composite of NaX and Nafion-Na, which enables the low-concentrated ASIBs with improved rechargeability, with 94.9% capacity retention after 200 cycles. The interaction between Nafion-Na and NaX regulates the self-assembled morphology of the interphase, giving shrunken ionic domains in the polymer matrix. Due to the size exclusion, the ion transport channels in Nafion-Na and cavities in NaX ensure rapid Na^+^ ion transport and provide a strong molecular sieving effect to reject hydrated Na^+^ ions, leading to the suppressed water-induced side reactions of even dilute aqueous electrolytes. The positive attributes concerning ESWs and full-cell cyclability provided by this interphase compare positively with those obtained from electrolyte modifications (such as using saturated 9 m NaOTF). Such a strategy can also be well applied to other low-concentrated aqueous electrolytes, such as 1 m Na_2_SO_4_, 2 m NaTFSI, and 2 m NaClO_4_, with similar superiority. More importantly, when combined with electrolyte modifications (e.g., WISE), the molecular-sieving interphase demonstrates the potential to further enhance the stability of ASIBs. This work offers opportunities for the development of other battery systems that face challenges related to solvent stability, such as those operating at high voltages.

## Supplementary Information

Below is the link to the electronic supplementary material.

Supplementary file1 (PDF 1707 kb)
