# Supplementary material for: A Molecular-Sieving Interphase Towards Low-Concentrated Aqueous Sodium-Ion Batteries
Source: Nanomicro Lett. 2024 Mar 4;16:144. doi: 10.1007/s40820-024-01340-5 (PMC10912067; doi:10.1007/s40820-024-01340-5)
Supplement: Supplementary file 1 — Supplementary file1 (PDF 1707 kb) [file 40820_2024_1340_MOESM1_ESM.pdf]

Supporting Information for

A Molecular-Sieving Interphase Towards Low-Concentrated Aqueous Sodium-Ion Batteries

Tingting Liu[1, 2, 4, 5, #], Han Wu[3, #], Hao Wang[1, 4, 5], Yiran Jiao[3], Xiaofan Du[1, 4, 5], Jinzhi Wang[1, 4, 5], Guangying Fu[1, 4, 5], Yaojian Zhang[1, 4, 5, *], Jingwen Zhao[1, 4, 5, *] and Guanglei Cui[1, 2, 4, 5, *]

[1] Qingdao Industrial Energy Storage Research Institute, Qingdao Institute of Bioenergy and Bioprocess Technology, Chinese Academy of Sciences, Qingdao 266101, P. R. China

[2] Center of Materials Science and Optoelectronics Engineering, University of Chinese Academy of Sciences, Beijing 100049, P. R. China

[3] School of Chemical Engineering and Advanced Materials, The University of Adelaide, Adelaide, SA 5005, Australia

[4] Shandong Energy Institute, Qingdao, 266101, P. R. China

[5] Qingdao New Energy Shandong Laboratory, Qingdao, 266101, P. R. China

[#] Tingting Liu and Han Wu contribute equally to this work.

*Corresponding authors. E-mail: zhangyj@qibebt.ac.cn (Yaojian Zhang); zhaojw@qibebt.ac.cn (Jingwen Zhao); cuigl@qibebt.ac.cn (Guanglei Cui)

Supplementary Tables and Figures

Table S1 Molecular dynamic (MD) simulations of NaOTF, and H_2O electrolytes

	NaOTf:H_2O =1.1:28
Number of NaOTF per box	88
Number of H_2O per box	2240
Total number of atoms	7512
Simulation box size (Å^3)	45.2×45.2×45.2
MD, density (g/cm^3)	1.168
Bias temperature (K)	298

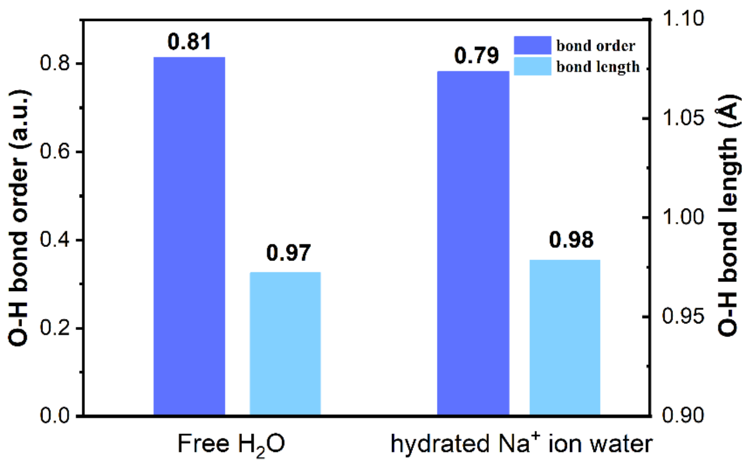

Fig. S1 Computed O-H bond order and bond length from solvation structures and free water molecules

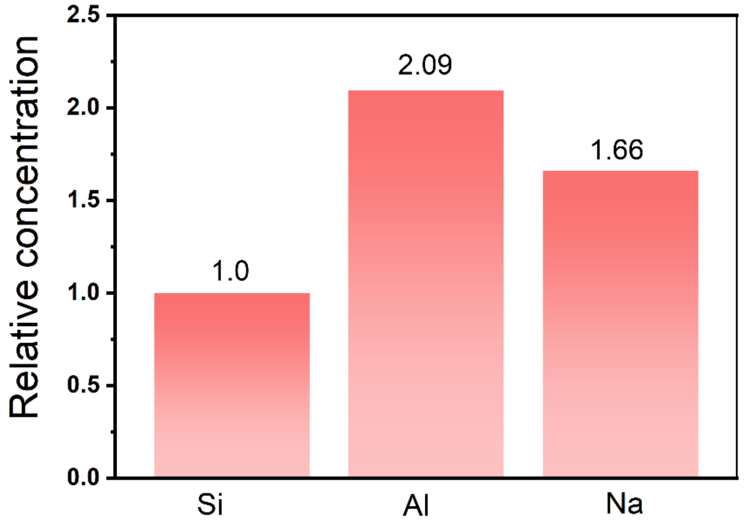

Fig. S2 ICP detection of NaX zeolite

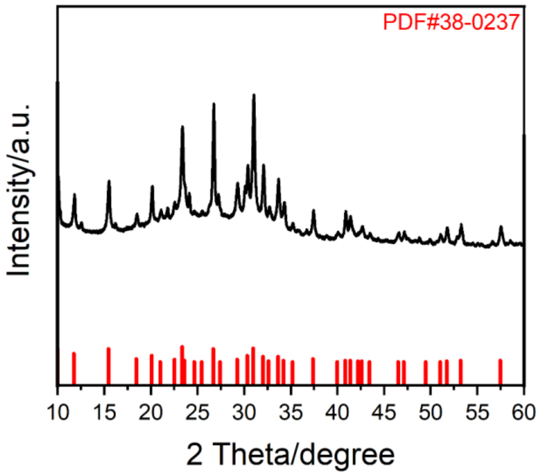

Fig. S3 XRD pattern of NaX zeolite

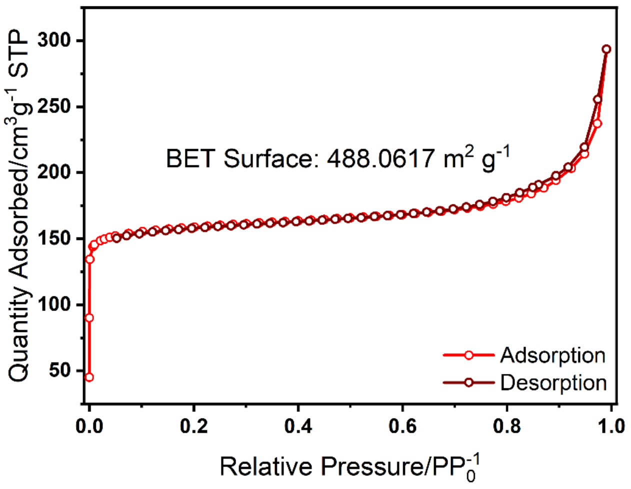

Fig. S4 N_2 adsorption/desorption isotherm of NaX zeolite

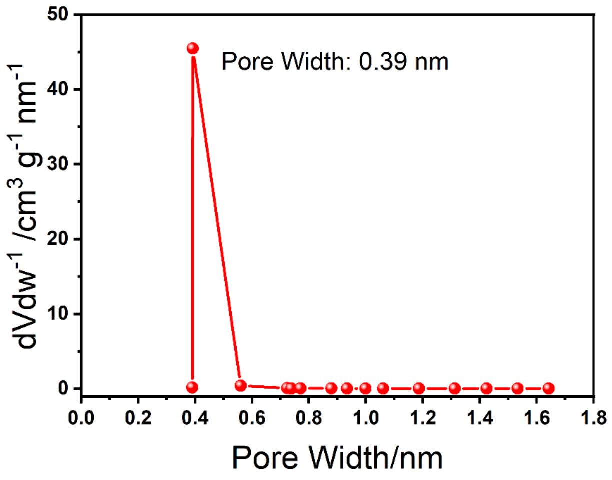

Fig. S5 Micropore volume dispersity of NaX zeolite

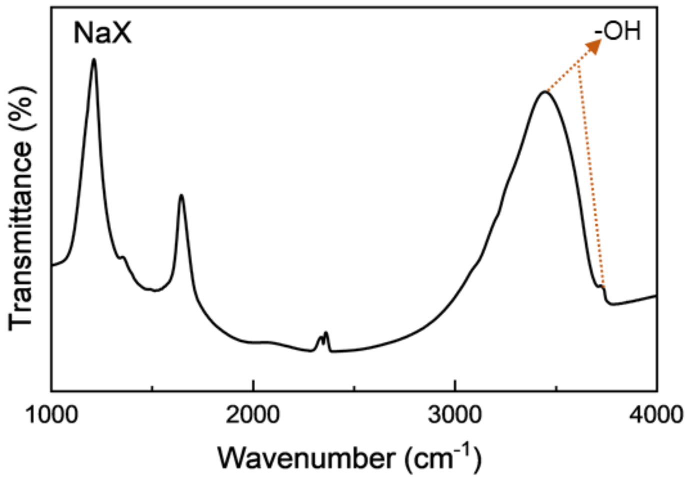

Fig. S6 The vacuum infrared spectrum of NaX

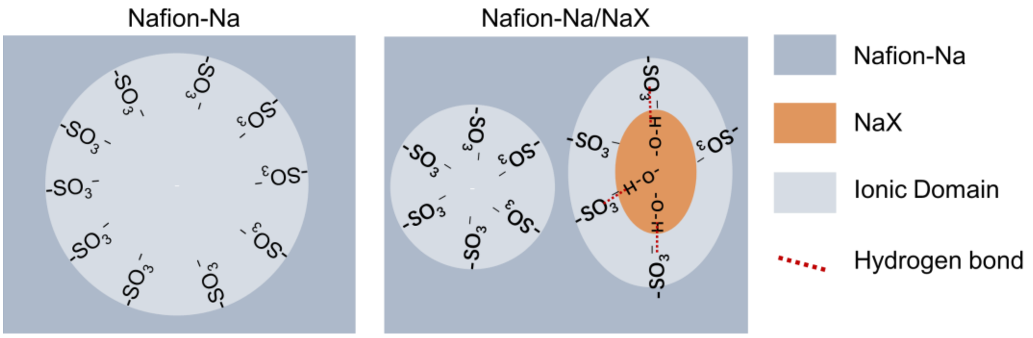

Fig. S7 Schematic diagram of the ion domain in Nafion-Na before and after the addition of NaX

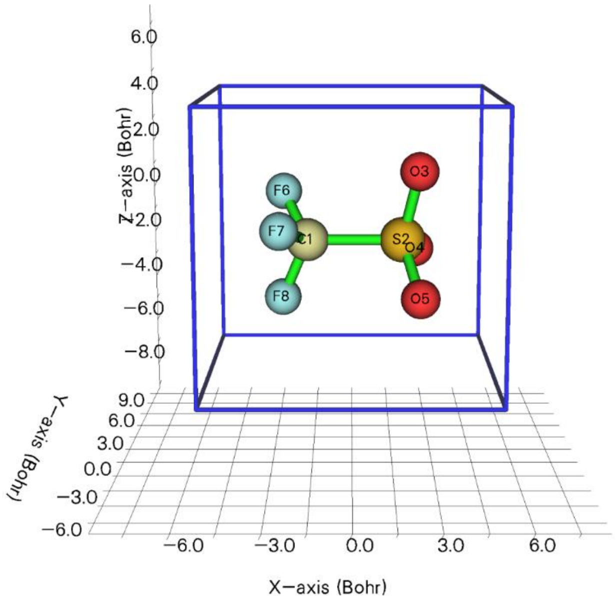

Fig. S8 The calculated Van der Waals diameter of OTf⁻ is 6.818Å

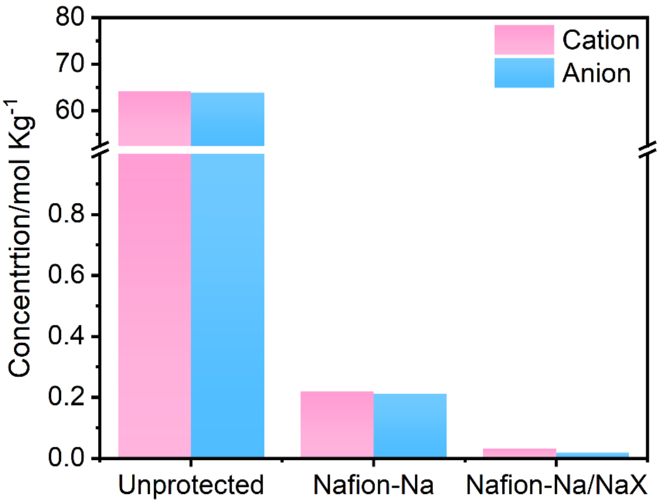

Fig. S9 ICP results of Na^+ and OTF^- permeability test with cellose, Nafion-Na coated cellose and Nafion-Na/NaX coated cellose membrane

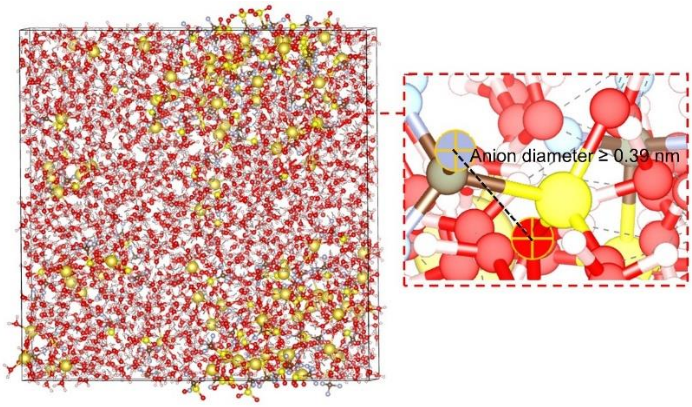

Fig. S10 Snapshot of MD simulation of 2 m NaOTF

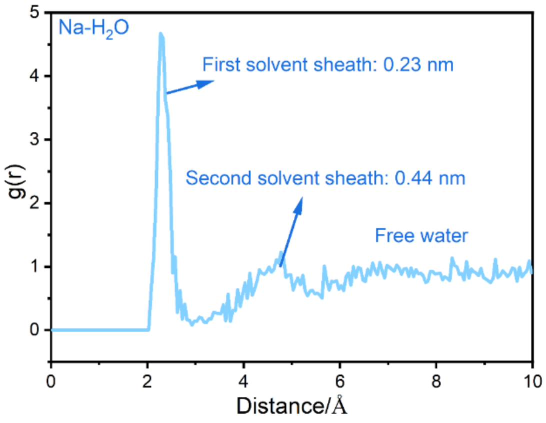

Fig. S11 MD simulations of electrolyte structure. The RDFs around Na^+ in 2 M NaOTF. The radial distance is the center of mass (COM) distance between center (Na^+) and H_2O molecules

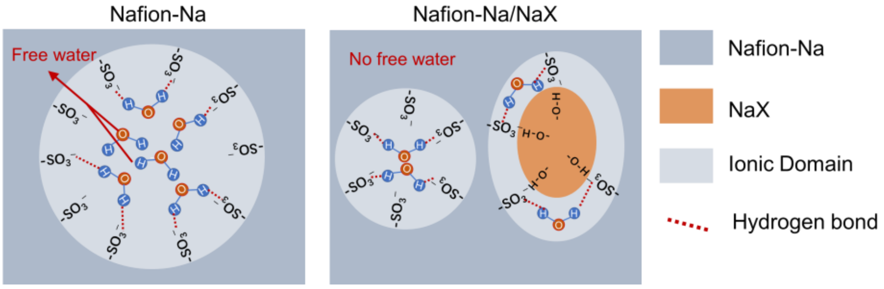

Fig. S12 Schematic diagram of the water state in Nafion-Na and Nafion-Na/NaX

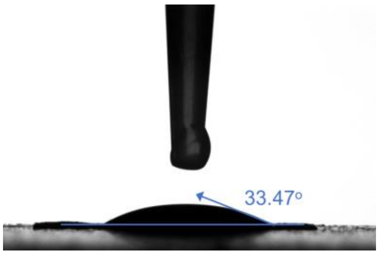

Fig. S13 The wetting behavior of NMF electrode surface with the precursor solution of Nafion-Na/Na

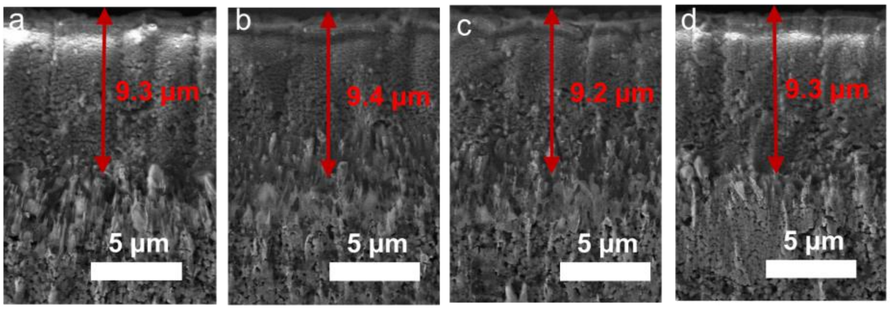

Fig. S14 SEM images of cross-sections at various locations of the NMF electrode coated with Nafion-Na/NaX

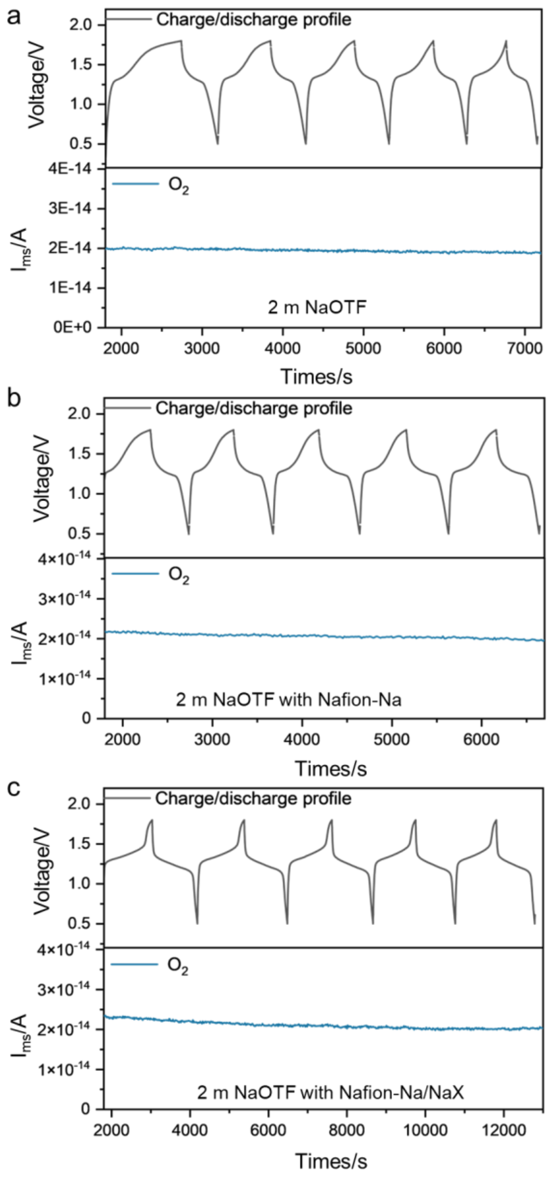

Fig. S15 Gas monitoring of NMF//NTP full cell in 2 m NaOTF. Potential and oxygen evolution as a function of time at 140 mA g^{-1} was monitored using a DEMS cell (after pre-cycled for 1 cycle) with (**a**) unprotected (**b**) Nafion-Na protected (**c**) Nafion-Na/NaX protected electrolytes, respectively

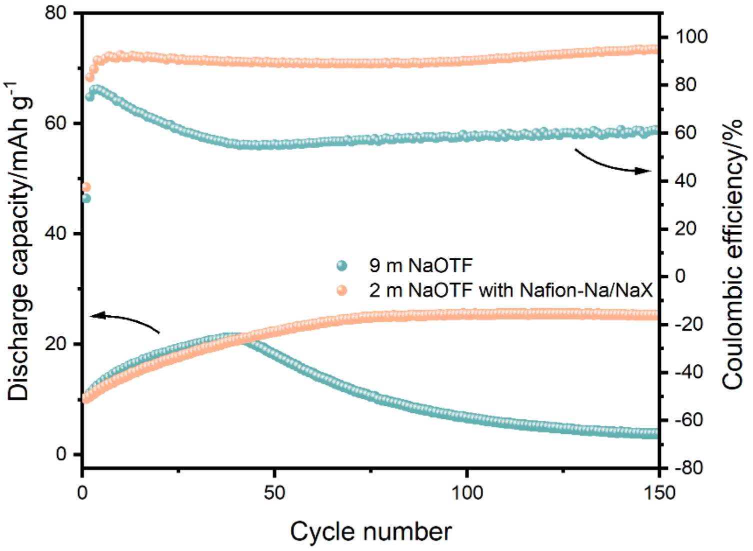

Fig. S16 Cycle performance comparion of NMF//NTP full cell with the unprotected 9 m NaOTF, 2 M NaOTF with Nafion-Na/NaX cycled at 140 mA g^{-1}

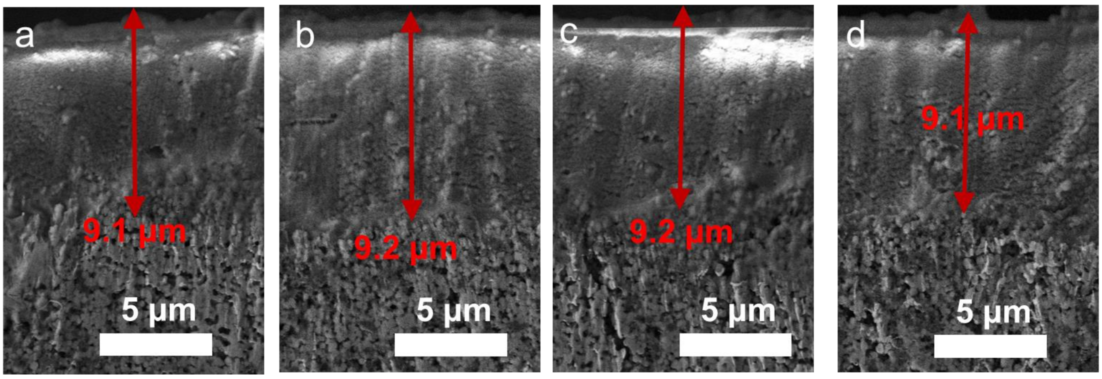

Fig. S17 SEM images of cross-sections of the NMF electrode coated with Nafion-Na/NaX after cycling

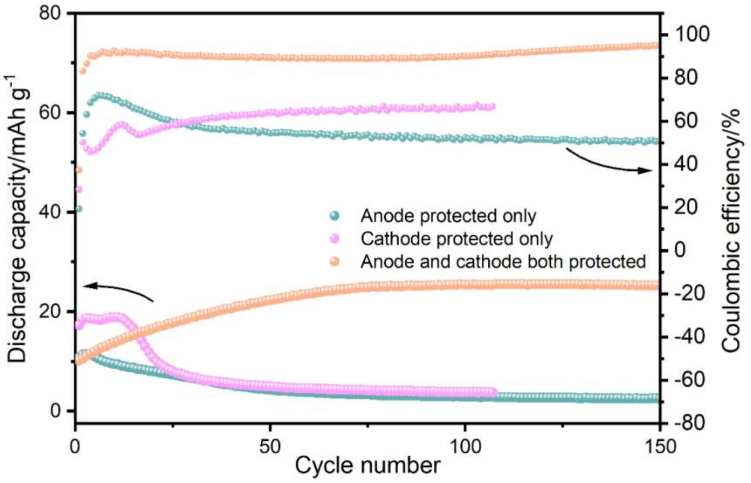

Fig. S18 Cycle performance comparion of the anode protected, cathdoe protected and fully protected NMF//NTP full cell

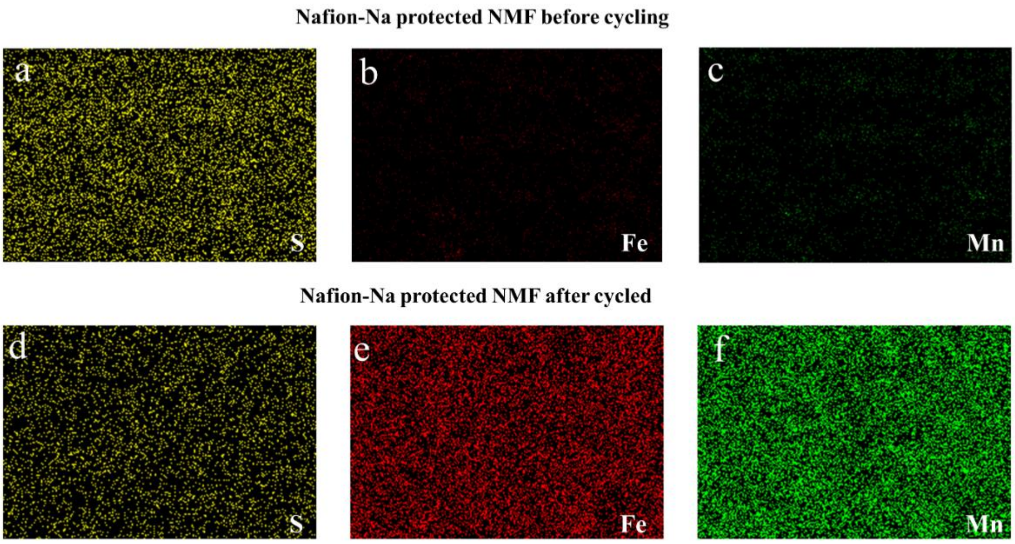

Fig. S19 EDS mapping of battery using Nafion-Na protected electrode in 2 m NaOTF electrolyte

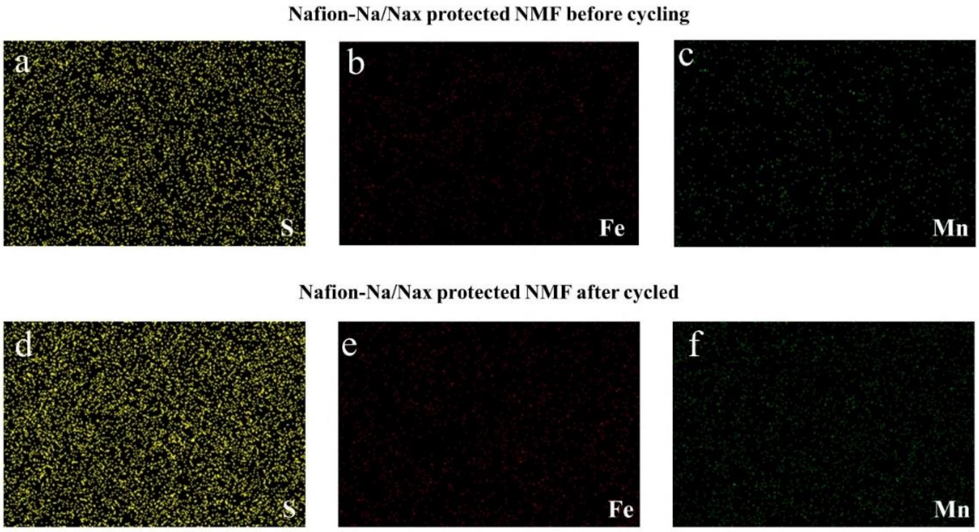

Fig. S20 EDS mapping of battery using Nafion-Na/NaX protected electrode in 2 m NaOTF electrolyte

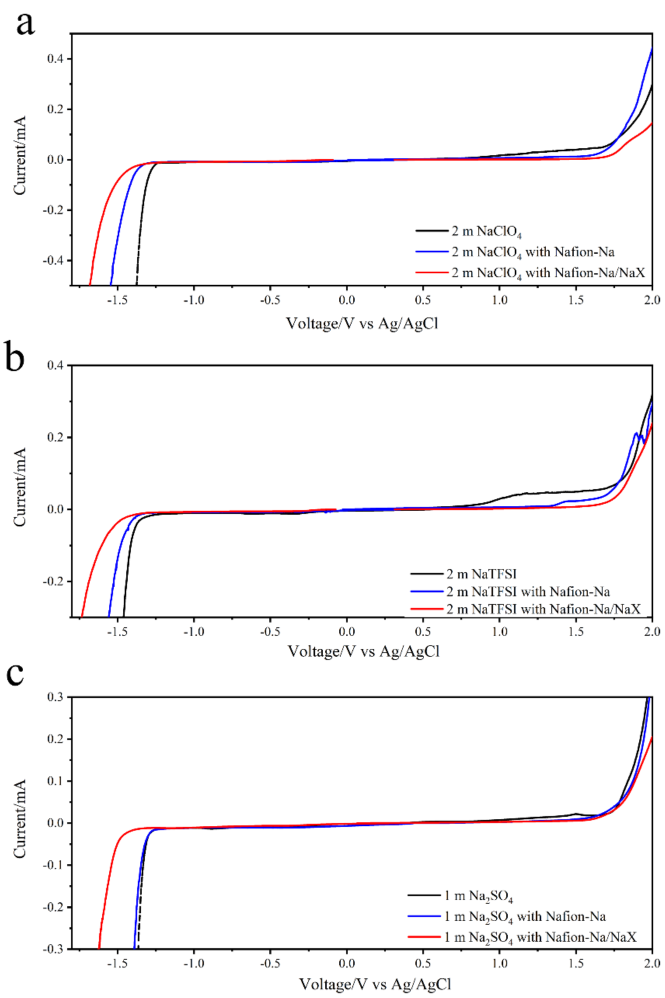

Fig. S21 The potential of hydrogen evolution and oxygen evolution on unprotected, Nafion-Na protected and Nafion-Na/NaX protected glassy carbon working electrode in (**a**) 2 m $NaClO_4$, (**b**) 2 m NaTFSI and (**c**) 1 m Na_2SO_4 electrolytes at a scan rate of 1 mV s^{-1}

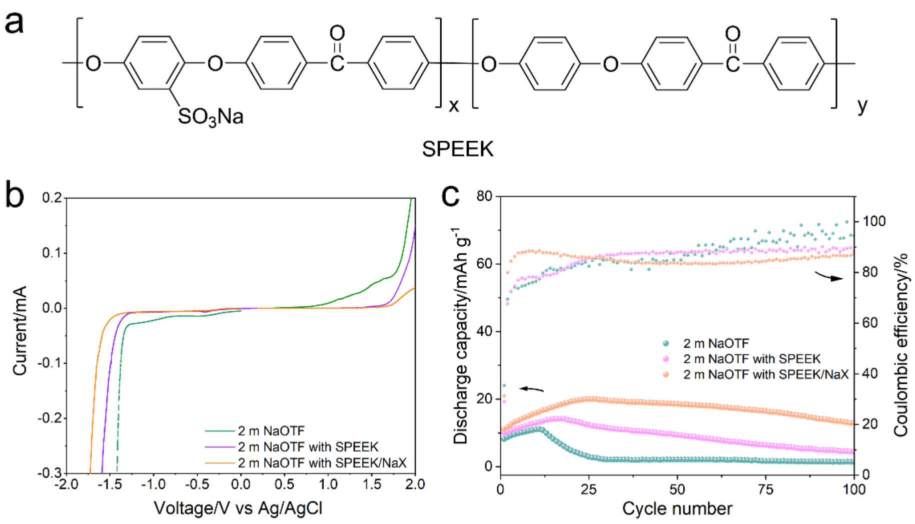

Fig. S22 (**a**) The chemical structure of SPEEK (**b**) Cycle performance comparion of the NMF/NTP full cell coupled with 2 m NaOTF at 140 mA g^{-1}

Table S2 The performance of reported electrode modifications for aqueous sodium ion batteries

Protective layer	Protected electrode	Electrolyte	Capacity Retention	Coulombic Efficiency (%)	References
70 nm HfO_2	$NaTi_2(PO_4)_3$	1 m Na_2SO_4	91% after 100 cycles (1 C)	94	[S1]
Polypyrrole	$NaTi_2(PO_4)_3$	1 m Na_2SO_4	-	95	[S2]
Polypyrrole	Pyromellitic dianhydride and 4,4'-oxydianiline	1 m Na_2SO_4	77.8% after 100 cycles (1 C)	-	[S3]
TiN	$NaTi_2(PO_4)_3$	1 m Na_2SO_4	69.7% after 100 cycles (2 C)	89	[S4]
Nafion-Na/NaX	$Na_2MnFe(CN)_6$// $NaTi_2(PO_4)_3$	2 m NaOTF	94.9% after 200 cycles (1 C)	96.8	This work

Supplementary References

[S1] L. Staisiunas, J. Pilipavicius, D. Tediashvili, J. Juodkazyte, L. Vilciauskas. Engineering of conformal electrode coatings by atomic layer deposition for aqueous Na-ion battery electrodes. J. Electrochem. Soc. **170**(5), 050533 (2023). https://doi.org/10.1149/1945-7111/acd4ee

[S2] W. Wang, J. Wu, C. Zeng. New construction of polypyrrole interphase layers to improve performance stability of $NaTi_2(PO_4)_3$ anode for aqueous na-ion batteries. Solid State Ion. **397**, 116259 (2023). https://doi.org/10.1016/j.ssi.2023.116259

[S3] B. Cho, H. Lim, H. N. Lee, Y. M. Park, H. Kim, H. J. Kim. High-capacity and cycling-stable polypyrrole-coated mwcnt@polyimide core-shell nanowire anode for aqueous rechargeable sodium-ion battery. Surf. Coat. Technol. **407**, 126797 (2021). https://doi.org/10.1016/j.surfcoat.2020.126797

[S4] Z. X. Liu, Y. F. An, G. Pang, S. Y. Dong, C. Y. Xu, C. H. Mi, X. G. Zhang. Tin modified $NaTi_2(PO_4)_3$ as an anode material for aqueous sodium ion batteries. Chem. Eng. J. **353**, 814 (2018). https://doi.org/10.1016/j.cej.2018.07.159
